# Oxidative stress and inflammatory markers in ovarian follicular fluid of women with diminished ovarian reserve during in vitro fertilization

**DOI:** 10.1186/s13048-023-01293-0

**Published:** 2023-10-23

**Authors:** Yan Huang, Yi Cheng, Min Zhang, Yan Xia, Xiaoyan Chen, Yexing Xian, Dewei Lin, Suyan Xie, Xinyu Guo

**Affiliations:** Center of Reproductive Medicine, the General Hospital of Southern Theater Command, Guangzhou, 510010 China

**Keywords:** Oxidative stress, Inflammation, Follicular fluid, Diminished ovarian reserve, TNF-α, IL-18, GSH

## Abstract

**Background:**

Follicular microenvironment has been proposed as an important factor for oocyte grown and maturation. We sought to evaluate the oxidative stress and inflammatory levels in follicular fluid (FF) and association with embryo quality in patients with diminished ovarian reserve (DOR).

**Methods:**

The current research included 46 DOR cases and 56 normal ovarian reserve (NOR) cases. Twelve representative oxidative stress markers and eight representative inflammatory factors were measured in the FF.

**Results:**

Oxidative stress markers total GSH (T-GSH) was decreased in the FF from women with DOR compared with that in NOR group (*P* = 0.041). More modest differences were observed for reduced GSH (rGSH) and rGSH/GSSG. Women with DOR compared to controls had higher level of TNF-α (*P* = 0.000) and lower level of IL-18 (*P* = 0.013). Correlation analysis revealed that GSSG was negatively correlated with normal fertilization rate in NOR group (r = -0.358, *P* = 0.008), and reduced GSH was negatively correlated with normal fertilization rate in DOR group (r = -0.299, *P* = 0.049). Moreover, as the regression analysis data showed, the GSSG level was significantly associated with embryo quality indicator.

**Conclusions:**

The FF in DOR patients was accompanied by increased oxidative stress and inflammatory levels. Follicular development of women with DOR might be influenced by unusual IL-18 and TNF-α levels in FF. And oxidative stress marker GSSG in NOR group was a negative predictor for embryo quality.

## Introduction

Ovarian reserve (OR) reflects the reproductive potential of women and refers to the number and quality of follicles at different development stages in the ovary. Diminished ovarian reserve is characterized by a decrease in basal antral follicle count (AFC) and anti-Müllerian hormone (AMH) and increased basal follicle stimulating hormone (FSH). Patients with DOR show impaired ovarian reserve, a risk of poor response to ovarian stimulation, fewer oocyte retrieved, and higher cycle cancellation rates, which dramatically reduces the chance of conceiving [1].

The etiological and pathological mechanisms of DOR are complex, mainly including age, genetic condition, immunity factor, chemoradiation and environment factors [2]. However, the primary cause is still difficult to determine.

Ovary follicle development is essential to oocyte competence for supporting embryonic development. The follicle comprises an antrum from the early antral follicle stage, and the follicular fluid in the antrum directly surrounds the oocyte and forms an important element of follicular microenvironment for oocyte grown and maturation. Follicular fluid is produced by secretions of the granulosa cells, theca cells and oocytes, and diffusion of plasma components across the blood-follicle barrier (BFB). The FF consists of a variety of lipids, microRNA, polysaccharides, cytokines, growth factors, inflammatory factors, reactive oxygen species and antioxidant enzymes [3–6]. The composition in FF reflects the follicular microenvironment surrounding the oocyte and is associated with follicle development and oocyte competence [7–9] and may be used to explore the mechanisms of various ovarian disorders.

Oxidative stress (OS) refers to an imbalance between reactive oxygen species (ROS) and antioxidative defense system, leading to DNA, proteins, and lipids oxidative damage. And OS plays an essential factor in ovarian aging [10]. The oxidative stress markers can be divided into two distinct phases: antioxidants including enzymatic and non-enzymatic molecules and oxidative damage to molecules. Reduced glutathione is a non-enzymatic antioxidant that neutralizes peroxides. Superoxide dismutase (SOD), catalase (CAT), glutathione peroxidase (GSH-PX), and peroxidase (POD) are representative enzymatic antioxidants. Total antioxidant capacity (TAOC), resulting from the enzymatic and non-enzymatic antioxidant defenses, represents the total antioxidant levels. Malondialdehyde (MDA) and lipid peroxidation (LPO) represent the final products of lipid peroxidation; protein carbonylation, is the major hallmark of protein oxidative damage; 8-Hydroxy-2’-deoxyguanosine (8-OHdG) is one of the predominant biomarkers of oxidative DNA damage. The oxidative stress levels in FF were examined in various studies involving different reproductive conditions during assisted reproductive techniques, including PCOS [11], endometriosis [12, 13] and obesity [14]. A systematic evaluation of oxidative stress marker in FF of DOR patients, on the other hand, has not been documented.

The concentrations of inflammatory factors in the follicular fluid affect oocyte maturation, follicular wall rupture, fertilization, and the development of early embryos [8, 15]. In the aging ovarian, the inflammatory reactions activated by NOD-like receptor 3 (NLRP3) and nuclear factor-kappa-gene binding (NF-κB) have been demonstrated in rodent models [16, 17], but less reported in human ovarian samples. NLRP3 causes a series of inflammatory reactions, such as the increased secretion levels of IL-1β and IL-18 [18]. Many molecules involved in the immune response and inflammatory response are regulated by NF-κB, which includes TNF-α, IL-1β, IL-2, IL-6 [18]. However, the concentrations of these inflammatory factors in FF of DOR patients are confused.

Currently, to our best knowledge, few studies focused on the inflammation and oxidative stress markers in FF of DOR patients. Liang et al. showed that 15 oxylipins metabolites was lower in the FF of DOR patients by UHPLC-MS-MS analysis [19]. Bouet et al. performed a targeted quantitative analysis of 27 cytokines and found Platelet-derived growth factor BB (PDGF-BB) was the only cytokine with a significantly lower concentration in the DOR group [20]. The concentrations of 480 cytokines and related growth factors in FF of DOR patients were determined using a multiplex immunoassay [21]. Fifty-nine cytokines had significantly different concentrations, which were less associated with NLRP3 and NF-κB signalings. Aim of the study was to determine the concentrations of oxidative stress markers, anti-inflammatory factors and pro-inflammatory factors in the FF of DOR patients undergoing the ovarian stimulation cycles, and compared them with NOR patients, in order to assess the impact of these factors on ovarian reserve.

## Materials and methods

### Study participants

We performed a prospective clinical study from February 2022 to June 2023. We enrolled a total of 102 patients under 40 years old with (n = 46) or without DOR (n = 56), who were undergoing IVF or ICSI treatment in the Centre for Reproductive Medicine, General Hospital of Southern Theatre Command. The study was approved by the Ethics Committees of General Hospital of Southern Theatre Command (Noc. NZLLKZ2022090). Before the study, all subjects signed informed consent.

The criteria for DOR included AMH, basal FSH, and bilateral AFCs [22, 23]. Patients were considered to have DOR when two of the following three criteria were present: bilateral AFC ≤ 7; AMH ≤ 1.1 ng/mL; and basal FSH on the second or third day of spontaneous menstrual cycle ≥ 10 IU/L. The NOR group included 56 infertile women who underwent IVF-ET treatment due to male factor, tubal factor and endometrial factor and had the serum AMH ≥ 1.5, 8 ≤ AFC ≤ 24 and FSH < 10 IU/L. Patients with radiotherapy, ovarian surgery, polycystic ovarian syndrome, polycystic ovaries, and endometriosis were excluded.

### Controlled ovarian stimulation (COS) protocol and embryo quality evaluation

Patients enrolled in our study received a standardized gonadotropin-releasing hormone (GnRH) antagonist or mild stimulation protocol for COS. When one or more follicles developed with diameters up to 20 mm, hCG was injected to induce follicular maturation and eggs were received by ultrasound-guided puncture 36 h later. In vitro fertilization (IVF) or intracytoplasmic sperm injection (ICSI) was performed based on the semen quality and previous fertilization history.

Fertilization was confirmed at 18-20 h after insemination by the presence of two pronuclei. Cleaved embryos were observed on day 3 after insemination. High-quality embryos were determined by blastomeres between 7 and 9 and the fragments ≤ 20%. Blastocyst morphological assessment were carried out on day 5 or day 6 by the development of cavity, trophoectoderm and inner cell mass (ICM). Rate of good quality embryos on day 3 was calculated as high-quality embryo number divided by 2PN zygotes. Blastocyst formation rate was calculated as blastocyst number divided by the embryo number of blastocyst culture.

### Serum hormone measurement and antral follicle calculation

Serum sex hormones (FSH, LH, E2, and P) and AMH were detected in all patients by radio-immunoassay and bilateral AFCs on menstrual cycle day 2 ~ 3 was calculated by transvaginal ultrasonography.

### Follicular fluid collection

Preovulatory follicular fluid was collected during oocyte retrieval. The FF was then centrifuged at 2000 rpm for 15 min at 4 °C to remove insoluble particles and cells. The supernatant was then transferred to a 2ml freezing tube, snap-frozen in liquid nitrogen and stored at -80 °C until assessment for:

Enzymatic and non-enzymatic antioxidants: rGSH, T-GSH, GSSG, GSH-PX, SOD, CAT, POD, and TAOC.

Molecules of oxidative damage: MDA, LPO, protein carbonylation, and 8-OHdG.

Inflammatory markers: IL-6, IL-18, TNF-α, IL-1β, high-sensitivity C-reactive protein(hs-CRP), IL-10, IL-2, and IL-15.

### Immunoassay and oxidative marker measurements

IL-10 (Cat# CSB-E04593h), IL-15 (Cat# CSB-E04603h), IL-18 (Cat# CSB-E07450h), IL-1β (Cat# CSB-E08053h), IL-2(Cat# CSB-E04626h), IL-6 (Cat# CSB-E04638h), TNF-α (Cat# CSB-E04740h), and hs-CRP (Cat# CSB-E08617h) were measured using a commercial ELISA kit (Cusabio, Wuhan, China). LPO (Cat# SEA296Hu) were determined by using an enzyme-linked immunosorbent assay kit (USCN Life Science Inc., Wuhan, China). 8-OHdG (Cat# ZC-31,674) determined by using an enzyme-linked immunosorbent assay kit (ZCIBIO Technology, shanghai, China).

Reduced GSH (Cat# A006-2-1), T-GSH (Cat# A061-1-2), GSSG (Cat# A061-1-2), GSH-PX (Cat# A005-1-2), SOD (Cat# A001-3-2), CAT (Cat# A007-1-1), TAOC (Cat# A015-2-1), MDA (Cat# A003-1-2), and Protein Carbonyl (Cat# A087-1-2) were all measured according to the manufacturer’s instructions (Nanjing Jiancheng Bioengineering Institute, China).

### Statistical analysis

The software SPSS version 21.0 (SPSS Inc., Chicago, IL, USA) was used to analyse the results. Normal distribution was tested by the Shapiro-Wilk test. The data were summarized using means ± standard deviations for continuous variables and frequencies and percentages for categorical variables. Differences between NOR and DOR were assessed through Student’s t-test for continuous variables and Mann–Whitney U test for categorical variables. The Spearman rank correlation coefficient was used for correlation analyses. To further explore which factors affected normal fertilization rate, multivariate stepwise regression analyses were performed to assess the effect of OS markers and inflammatory factors on normal fertilization rate. Statistical significance was defined as *P* ≤ 0.05.

## Results

### Baseline characteristics of the DOR and NOR groups

A total of 102 samples including 46 patients with DOR and 56 patients with NOR were obtained. The backgrounds of the DOR and NOR groups are summarized in Table 1. Patients with DOR were older and had higher levels of basal FSH and total dosage of FSH for ovarian stimulation than patients in NOR group. The bilateral basal AFC, AMH, number of oocytes retrieved, and number of MII oocytes were significant lower in DOR group compared with NOR group.

**Table 1:** Baseline indicators of patients with DOR and NOR

	NOR (n = 56)	DOR (n = 46)	*P*
Female age (y)^&^	32.46 ± 3.62	34.95 ± 4.33	0.005^*^
Male age(y)^&^ Bilateral basal AFC^#^ BMI (kg/m^2^)^&^ AMH (ng/ml)^#^ Basal FSH^#^ E2 (pg/ml)^#^ LH (mIU/mL)^#^ P (ng/ml)^#^ Total FSH used (IU)^#^ Days of stimulation Number of oocytes retrieved^#^ Number of MII oocytes^#^	33.82 ± 3.75 15.0 (11.0-21.75) 22.37 ± 2.86 3.47(2.03–4.37) 6.40(5.40–7.50) 139.05(97.95–170.27) 52.81(19.07–79.07) 0.88 (0.56–1.22) 1800(1540–2250) 9.38 ± 2.56 15.0 (11.0–21.0) 12.0 (9.0–18.0)	35.58 ± 4.05 5.0(4.0–7.0) 21.68 ± 3.03 0.77(0.49–1.02) 8.8(7.15–11.35) 145.5(115.7–193.17) 77.07(32.89–122.60) 0.89(0.60–1.37) 2700(1950–3206) 9.58 ± 3.20 3.0 (2.0–5.0) 2.0 (2.0–4.0)	0.026^*^ 0.000^*^ 0.249 0.000^*^ 0.000^*^ 0.041^*^ 0.005^*^ 0.947 0.000^*^ 0.891 0.000^*^ 0.000^*^
Normal fertilization (%)^&^	65.56 ± 22.38	67.10 ± 31.60	0.640
High quality embryo (%)^#^	30.95(12.50–65.90)	33.33(11.30-54.16)	0.380^*^

DOR, Diminished ovarian reserve; NOR, Normal ovarian reserve; AFC, antral follicle count; BMI, Body mass index; AMH, anti-Müllerian hormone;

FSH, follicle-stimulating hormone; LH, luteinizing hormone; E2, estrogen; P, progesterone.

*Difference is considered significant.

^&^Values are presented as the mean ± SD for continuous indicators following normal distribution. Student’s t-test for continuous variables

^#^ Values are presented as the median (interquartile range) for continuous indicators not following normal distribution. Mann–Whitney U test for categorical variables

### Oxidative stress and inflammatory levels in the DOR and NOR group

After adjustment for age and dosage of FSH for ovarian stimulation, in women with DOR, we found lower values of T-GSH [10.84(9.63–12.53) vs. 9.90(9.63–12.01), *P*adj = 0.030] (Table 2) in the follicular fluid compared with the NOR group. No significant differences between groups were observed in rGSH, GSSG, GSH-PX, SOD, POD, CAT, TAOC, MDA, LOP, protein carbonylation and 8-OHdG.

**Table 2:** Oxidative stress markers in the FF of patients with DOR and NOR

	Control	DOR	*P*	*P* adjusted
rGSH (µmol/L)^#^	5.81(3.48–7.22)	4.65(2.86–6.63)	0.117	0.178
T-GSH (µmol/L)^#^	10.84(9.63–12.53)	9.90(9.63–12.01)	0.041^*^	0.030^*^
GSSG (µmol/L)^#^ rGSH/GSSG^&^ GSH-PX (U/mL)^&^ SOD (U/mL)^#^ POD (U/mL)^#^ CAT (U/mL)^&^ TAOC (U/mL)^#^ MDA (nmol/mL)^#^ LPO (µmol/L)^#^	4.69(4.64–6.57) 1.21 ± 0.90 148.97 ± 39.34 31.28(30.50-32.74) 7.93(7.03–9.05) 3.14 ± 1.22 0.97(0.88–1.04) 2.12(1.63–2.79) 40.72(34.43–44.59)	4.69(4.63–5.63) 1.02 ± 0.52 141.07 ± 50.15 31.57(29.55–32.97) 8.33(6.46–10.16) 3.08 ± 0.97 1.00(0.91–1.09) 2.19(1.52–2.63) 40.72(36.82-46.00)	0.888 0.208 0.390 0.996 0.672 0.455 0.366 0.668 0.932	0.349 0.380 0.554 0.659 0.164 0.454 0.568 0.856 0.298
Protein Carbonylation (nmol/mgprot)^#^	0.53(0.23–0.81)	0.56(0.34–0.86)	0.227	0.325
8-OHdG (ng/mL)^#^	57.49(32.18–78.95)	55.23 (35.16–82.52)	0.156	0.316

rGSH, reduced glutathione; T-GSH, total glutathione; GSSG, oxidized glutathione; GSH-PX, Glutathione peroxidase; SOD, superoxide dismutase; POD, peroxidase; CAT, catalase; TAOC, total antioxidant capacity; MDA, malondialdehyde; LPO, lipid peroxidation; 8-OHdG, 8-Hydroxy-2’-deoxyguanosine.

*Difference is considered significant.

^&^Values are presented as the mean ± SD for continuous indicators following normal distribution. Student’s t-test for continuous variables

^#^ Values are presented as the median (interquartile range) for continuous indicators not following normal distribution. Mann–Whitney U test for categorical variables\

*P* adjusted: age and dosage of FSH for ovarian stimulation are adjusted as covariants.

Follicular fluid concentrations of IL-6, IL-18, TNF-α, IL-1β, hs-CRP, IL-10, IL-2, and IL-15 in patients with DOR and NOR group are presented in Table 3. The level of proinflammatory cytokine TNF-α in DOR group was much higher than that in NOR group [58.92(41.95–81.59) vs. 92.24(64.30-126.60), *P*adj = 0.003]. Furthermore, median level of cytokine IL-18 in the DOR group was lower than that in NOR group [25.47(4.33–50.71) vs. 10.65(3.86–24.71), *P*adj = 0.05].

**Table 3:** Inflammatory factors in the FF of patients with DOR and NOR

	Control	DOR	*P*	*P* adjusted
IL-6 (pg/mL)^#^	1.89 (0.79–5.25)	1.89(0.91–8.41)	0.528	0.414
IL-18 (pg/mL)^#^	25.47(4.33–50.71)	10.65(3.86–24.71)	0.013*	0.05*
TNF-α (pg/mL)^#^ IL-1β (pg/mL)^#^ hs-CRP (ng/mL)^#^ IL-10 (pg/mL)^#^ IL-2 (pg/mL)^#^ IL-15 (pg/mL)^&^	58.92(41.95–81.59) 1.29(0.75–4.08) 744.68(289.69-1498.96) 3.32(1.79–5.04) 5.99(3.12–11.70) 3.91 ± 2.96	92.24(64.30-126.60) 1.84(1.04–5.64) 689.04(232.28-1218.75) 4.13(1.40-12.94) 4.46(2.96–10.77) 4.28 ± 3.19	0.000* 0.298 0.650 0.258 0.236 0.568	0.003* 0.359 0.060 0.069 0.929 0.551

IL-6, interleukin 6; IL-18, interleukin 18; TNF-α, tumor necrosis factor α; IL-1β interleukin 1β; hs-CRP, high-sensitivity C-reactive protein; IL-10, interleukin 10; IL-2, interleukin 2; IL-15, interleukin 15; IL-8, interleukin 8.

*Difference is considered significant.

^&^Values are presented as the mean ± SD for continuous indicators following normal distribution. Student’s t-test for continuous variables

^#^ Values are presented as the median (interquartile range) for continuous indicators not following normal distribution. Mann–Whitney U test for categorical variables

*P* adjusted: age and dosage of FSH for ovarian stimulation are adjusted as covariants.

### Oxidative stress, inflammation, and embryo quality

High-quality oocytes or embryos indicate good pregnancy outcomes. The normal fertilization rate and day 3 high-quality embryo rate are usually indicators of oocyte or embryo quality. The normal fertilization rate and high-quality embryo rate were similar between DOR and NOR group (Table 1). Correlation analysis revealed that GSSG was negatively correlated with normal fertilization rate in NOR group (r = -0.358, *P* = 0.008). Furthermore, Reduced GSH was negatively correlated with normal fertilization rate in DOR group (r = -0.299, *P* = 0.049). Interesting, we did not find any inflammatory factors correlated with normal fertilization rate or day 3 high-quality embryo rate in DOR and NOR group, respectively.

In multivariate stepwise regression analysis, other embryo quality-related indicators, including age, total dosage of FSH for ovarian stimulation, BMI, E2, P, basal FSH, LH, AMH were analyzed with GSSH and rGSH, respectively. As shown in Table 4, GSSG and E2 were significant factors affecting the normal fertilization rate in NOR group. In the DOR group, LH and BMI were significant factors affecting the normal fertilization rate.

**Table 4:** Correlation coefficients between oxidative stress markers and normal fertilization

Independents	Unstandardized coefficients			Standardized coefficients	*t*	*P*
	*B*	SE		Beta		
Model I: In DOR group normal fertilization rate as dependent						
Constant	-13.221	31.812			-0.416	0.680
BMI	4.157	1.498		0.391	2.775	0.008
LH	-3.894	1.403		-0.391	-2.775	0.008
Model II: In NOR group normal fertilization rate as dependent						
Constant	83.422	10.172			8.203	0.000
E2	-0.016	0.006		-0.318	-2.514	0.015
GSSG	-4.467	1.778		-0.318	-2.514	0.015

## Discussion

In this study, we simultaneously measured the oxidative stress and inflammatory markers in the follicular fluid of DOR and NOR group, and evaluated the relationship between these makers with embryo quality. We demonstrated that FF in DOR patients had higher oxidative stress and inflammatory levels which might be closely related to the pathogenesis of DOR. Furthermore, oxidative stress marker GSSG in FF was founded to be closely correlated with normal fertilization rate in NOR group.

The oxidant–antioxidant state of follicular fluid and its effects on oocyte and IVF outcomes has been of great interest in recent years. Oxidative stress levels in the FF exerts a local and direct influence on the microenvironment for oocyte development. The oxidative stress markers in FF were examined in various studies involving different reproductive conditions during assisted reproductive techniques, including PCOS [11], endometriosis [12, 13] and obesity [14], whereas few of studies examined the changes of oxidative stress levels in DOR. In this study, we examined the levels of enzymatic and non-enzymatic antioxidants, including rGSH, GSSG, T-GSH, rGSH/GSSG, SOD, CAT, GSH-PX, and POD, and molecules of oxidative damage, including MDA and LPO, protein carbonylation, and 8-OHdG, final products of lipid peroxidation, the major hallmark of protein oxidative damage and the predominant biomarker of oxidative DNA damage, respectively. Importantly, TAOC, the total antioxidant levels and resulting from the enzymatic and non-enzymatic antioxidant defenses, was also measured in our study. Therefore, we provided a rigorous and comprehensive evaluation of oxidative stress levels in FF of patients with DOR.

In the present study, T-GSH levels were lower in DOR group. GSH is very important in oocyte maturation and fertilization process, including oocyte spindle function and pronucleus development, respectively [24]. Reduced GSH could directly scavenge the free radicals and oxidized to the GSSG. T-GSH is the sum of GSSG plus rGSH. In the cumulus cells of PCOS patients, the ratio of rGSH/GSSG was lower, suggesting enhanced oxidative stress [25]. Our results showed that T-GSH level is lower in patients with DOR, and the rGSH and rGSH/GSSG were simultaneously decreased in DOR group. These results suggested increased oxidative stress in FF of patients with DOR.

Oxidative stress is closely linked with inflammation. The redox imbalance in the ovarian could trigger the assembly and activation of NLRP3, an inflammasome in the NLR family. After activation of NLRP3 inflammasome, the secretion of pro-inflammation cytokines IL-1β and IL-18 were increased. ROS can also directly activate NF-κB to promote inflammation while activation of NF-κB further upregulates NLRP3 and TNF-α expression. NLRP3 was found to be highly expressed in the granulosa cells of DOR patients [16]. After NLRP3 reduction, the levels of pro-inflammatory cytokines were down-regulated while the levels of AMH and the number of primordial follicles increased in a rodent model [16].

In our study, we checked the concentrations of IL-1β and IL-18. IL-1β was elevated in DOR group but without statistically significant. A previous study showed that the level of IL-1β was significantly higher in the ovaries of aged mice than in younger mice [26]. IL-1β could damage the ovarian reserve through inflammation caused by inhibiting autophagy [27].

Yet the concentration of IL-18 was lower in the group of patients with DOR than that in NOR group. Tsuji et al. demonstrated IL-18 receptor blockade by IL-18R monoclonal antibody reduced the number of ovulated oocytes and inhibited the expansion of cumulus cells surrounding the ovum [28]. It has been demonstrated that IL-18 could directly affect the function of bovine theca cells, including promotion of cell proliferation and steroidogenesis [29]. In this study, we found IL-18 was lower in FF of patients with DOR, which permitted speculation about the decrease of IL-18 in FF might affect the function of theca cells and follicular development, leading to the defects of follicular development.

Plasma levels of TNF-α were increased in patients with DOR compared to healthy controls [16]. And the mouse model have shown that genetic deletion of TNF-α were associated with increased proliferation of granulosa cells, decreased apoptosis of oocytes and increased follicular reserve at aged mice [30], meanwhile intra-ovarian mRNA level of TNF-α was significantly increased during ovarian ageing in mice [31]. There was an increased level of TNF-α in FF of patients with PCOS compared with control group [32], indicating the association between PCOS and low-grade chronic inflammation. In our study, we also found a significantly increased TNF-α level in the FF of DOR patients, implying an inflammatory microenvironment during the follicular development.

Besides inducing inflammatory responses, TNF-α had significant inhibitory effects on steroidogenesis and folliculogenesis. Rice et al. demonstrated TNF-α inhibited the estradiol secretion induced by FSH in human granulosa cell [33]. Meanwhile, using rat granulosa cells, Darbon et al. showed TNF-α might reduce the granulosa cell differentiation induced by FSH and LH during the physiological ovarian follicular maturation [34]. The negative effect of TNF-α on steroidogenesis and folliculogenesis might be associated with reduced ovarian reserve in DOR patients.

We found a statistically significant negative correlation between GSSG in the FF with normal fertilization rate in NOR group. GSSG production is involved the oxidation of reduced GSH during the detoxification of reactive oxygen species. So, these findings were not surprising and might have a logical explanation. Similarly, we found GSH was negatively correlated with normal fertilization rate in DOR group. The lower concentrations of GSH in FF might reflect excess consumption to neutralize ROS, and the FF with high concentration of GSH might be an oxidative stress condition.

Our results indicated that normal fertilization rate and high-quality embryo rate were not decreased in DOR patients, which indicated that DOR had little influence on the quality of oocytes and embryos. The result of the present study was agreement with precious study, which showed DOR was not associated with a decreased oocyte quality [35, 36].

## Conclusions

To the best of our knowledge, this is the first study exploring the changes of oxidative stress and inflammatory levels in FF of patients with DOR compared with patients with NOR. The results showed that FF in DOR patients were associated with increased oxidative stress and inflammatory markers. It was shown that reduced antioxidant status and increased inflammatory level in the FF are characteristics of DOR patients. This is firstly demonstrated the reduced IL-18 level in FF of patients with DOR compared with patients with NOR. We proposed that IL-18 and TNF-α was associated with steroidogenesis and folliculogenesis, and the function of them remains to be furtherly studied to document its significance in the pathophysiology of DOR.

## Data Availability

The data sets analysed during the current study are available from the corresponding author on reasonable request.

## Abbreviations

DOR: Diminished ovarian reserve
NOR: Normal ovarian reserve
AFC: antral follicle count
BMI: Body mass index
AMH: anti-Müllerian hormone
FSH: follicle-stimulating hormone
LH: luteinizing hormone
E2: estrogen
P: progesterone
rGSH: reduced glutathione
T-GSH: total glutathione
GSSG: oxidized glutathione
GSH-PX: glutathione peroxidase
SOD: superoxide dismutase
POD: peroxidase
CAT: catalase
TAOC: total antioxidant capacity
MDA: malondialdehyde
LPO: lipid peroxidation
8-OHdG: 8-Hydroxy-2’-deoxyguanosine
IL-6: interleukin 6
IL-18: interleukin 18
TNF-α: tumor necrosis factorα
IL-1β: interleukin 1β
hs-CRP: high-sensitivity C-reactive protein
IL-10: interleukin 10
IL-2: interleukin 2
IL-15: interleukin 15
